# Using Tox21 High-Throughput Screening Assays for the Evaluation of Botanical and Dietary Supplements

**DOI:** 10.1089/aivt.2018.0020

**Published:** 2019-03-09

**Authors:** Troy D. Hubbard, Jui-Hua Hsieh, Cynthia V. Rider, Nisha S. Sipes, Alexander Sedykh, Bradley J. Collins, Scott S. Auerbach, Menghang Xia, Ruili Huang, Nigel J. Walker, Michael J. DeVito

**Affiliations:** ^1^ Division of the National Toxicology Program, National Institute of Environmental Health Sciences, Research Triangle Park, North Carolina.; ^2^ Kelly Government Solutions, Durham, North Carolina.; ^3^ Sciome, Durham, North Carolina.; ^4^ National Center for Advancing Translational Sciences, National Institutes of Health, Rockville, Maryland.

**Keywords:** botanical supplements, dietary supplements, *in vitro* screening, Tox21, toxicity

## Abstract

***Introduction:*** Recent nationwide surveys found that natural products, including botanical dietary supplements, are used by ∼18% of adults. In many cases, there is a paucity of toxicological data available for these substances to allow for confident evaluations of product safety. The National Toxicology Program (NTP) has received numerous nominations from the public and federal agencies to study the toxicological effects of botanical dietary supplements. The NTP sought to evaluate the utility of *in vitro* quantitative high-throughput screening (qHTS) assays for toxicological assessment of botanical and dietary supplements.

***Materials and Methods:*** In brief, concentration–response assessments of 90 test substances, including 13 distinct botanical species, and individual purported active constituents were evaluated using a subset of the Tox21 qHTS testing panel. The screen included 20 different endpoints that covered a broad range of biologically relevant signaling pathways to detect test article effects upon endocrine activity, nuclear receptor signaling, stress response signaling, genotoxicity, and cell death signaling.

***Results and Discussion:*** Botanical dietary supplement extracts induced measurable and diverse activity. Elevated biological activity profiles were observed following treatments with individual chemical constituents relative to their associated botanical extract. The overall distribution of activity was comparable to activities exhibited by compounds present in the Tox21 10K chemical library.

***Conclusion:*** Botanical supplements did not exhibit minimal or idiosyncratic activities that would preclude the use of qHTS platforms as a feasible method to screen this class of compounds. However, there are still many considerations and further development required when attempting to use *in vitro* qHTS methods to characterize the safety profile of botanical/dietary supplements.

## Introduction

Use of dietary supplements in support of maintaining a healthy lifestyle or for purported medicinal benefits is becoming increasingly prevalent among American consumers. Sales of herbal dietary supplements in the United States increased by 8.5% in 2017, with consumers spending over $8 billion.^1^ According to data collected by the National Health and Nutrition Examination Survey (NHANES) from 2011 to 2012, 52% of U.S. adults reported use of a dietary supplement within the preceding 30 days.^2^ Further data reported in the 2012 National Health Information survey found 18% of respondents used nonvitamin/nonmineral dietary supplements, such as fish oil, probiotics, and/or botanicals.^3^

Dietary supplements are defined under the Dietary Supplement Health and Education Act of 1994 (DSHEA) as a food, although “not represented for use as a sole item of a meal or of the diet,” and must contain one or more dietary ingredients, such as vitamins, minerals, amino acids, or herbs or other botanicals, and must be intended for ingestion.^4,5^ The federal regulatory framework instilled by DSHEA ensures consumer access to dietary supplements and establishes guidelines for good manufacturing practices for the industry. Botanical dietary supplements may be composed of whole plants, plant parts, dried/powdered plants, or plant extracts. These botanicals are produced and sold in various forms such as powders, capsules, essential oils, tinctures, and teas, and are present as a component in a formulation that may contain other botanicals or bioactive ingredients, as well as excipients.

There exists a prevailing public perception that dietary supplements derived from “natural” ingredients are inherently nontoxic; however, multiple reports of adverse events in humans have been associated with use of botanical dietary supplements.^6^ In addition to the potential for plant constituents to elicit toxicity, dietary supplements may be adulterated with pharmaceutical agents or contain contaminants (e.g., pesticides, mycotoxins, and heavy metals) that are potentially toxic.^7–12^ Ultimately, the burden of proof is placed on the FDA to monitor reports of adverse events associated with dietary supplement use and to demonstrate evidence that a product is unsafe or adulterated to restrict its use or remove it from the market.

The FDA faces numerous challenges in regulating botanical dietary supplements, including complexity of product formulations, variability in the chemical composition of botanical source materials, and adulteration of products.^13^ Recommended dose, phytochemical levels, suggested durations of use, and compliance with label recommendations vary widely and can contribute to high exposure levels and prolonged use among consumers. Regulations concerning premarket safety of dietary supplements are distinct from those for pharmaceuticals in that manufacturers are not required to provide the FDA with efficacy data or safety data before marketing the product if there is sufficient evidence that dietary ingredients were marketed or present in the food supply before 1994. In some cases, historical information may not be sufficient to clearly establish safety. This has led to numerous nominations to the National Toxicology Program (NTP) to study the potential harm of short- and long-term exposure to botanical dietary supplements.^14^

In 2012, the FDA estimated a total of 55,600 dietary supplements were readily available to consumers and ∼5560 new dietary supplement products enter the market each year.^15^ Due to the cost, duration, and ethical concerns surrounding animal testing, it is not a feasible primary screening method to address potential safety concerns. *In vitro* quantitative high-throughput screening (qHTS) methods could provide an efficient means to evaluate hazard/toxicity of botanical dietary supplements. The Toxicology in the 21st Century (Tox21) federal collaboration has developed a high-throughput *in vitro* testing program to evaluate potential human toxicities of thousands of chemicals. This testing program is composed of greater than 60 qHTS assays conducted in human cell lines that encompass a broad range of toxicologically relevant pathways and endpoints to identify potential biological targets or adverse outcomes (e.g., cytotoxicity, cellular stress, mitochondrial function, and nuclear receptor binding and activity).^16^ Data generated by the Tox21 testing program are publicly available and have been used to prioritize remediation efforts for hazardous substances found in Superfund sites and to support cancer hazard evaluations.^17,18^ Unlike single-chemical test agents, botanical supplements are complex mixtures that may exhibit compositional variability due to natural variation in source material and differences in manufacturing processes, as well as the possibility of contamination and/or adulteration. Approaches that attempt to characterize potential hazard by quantification and assessments of individual chemical constituents may not adequately capture the biological activity profiles associated with botanical mixtures/formulations. Although botanical/dietary formulations are marketed as bioactive substances, it is not known whether this activity can be measured using Tox21 *in vitro* qHTS assays, or how this activity compares to the entirety of the chemical space previously analyzed in the Tox21 program.

Botanical/dietary supplements present a formidable challenge to traditional methods for safety and hazard evaluations due to their chemical complexity, variability among marketed formulations, and the inability to test every marketed formulation, available lot, or presumed bioactive constituent. Therefore, the NTP sought to investigate if the Tox21 *in vitro* qHTS assay panel could be used to evaluate the mechanistic and biological activity of complex botanical mixtures. Herein, we describe the relative biological activities of dietary/botanical formulations in a subset (20 endpoints) from the Tox21 *in vitro* testing program. These data serve to characterize biological activity across an array of botanical/dietary supplements and to compare differential activity between individual marker constituents and their respective botanical. The distribution of biological activity for botanical/dietary substances was then compared to that of thousands of previously analyzed chemicals in the Tox21 10k library. In summary, this proof-of-concept study provides novel insight to the concept of biological fingerprinting as a means to evaluate complex botanical/dietary mixtures and identifies numerous challenges and considerations for future translational use.

## Materials and Methods

### Test substance preparation and plate generation

Botanical and dietary test substances were selected based upon availability due to previous procurement for pending, ongoing, or previously completed studies as part of the NTP Botanical Dietary Supplements Research Program. A comprehensive inventory of tested materials can be seen in Supplementary Table S1. Extracts of dietary supplements and constituents were prepared by weighing out 10 mg of test material and adding 1 mL of dimethyl sulfoxide (DMSO) (Sigma Aldrich) and vortexing samples at high speed for ∼2 minutes. After vortexing, extracts were centrifuged for 5 minutes at (∼16,000 *g*). Supernatant was removed and aliquots (50 μL) of each preparation were transferred to six replicate 384-well plates. Multiple lots were prepared for some formulations and constituents. Annatto, citral, curcumin, *Ginkgo biloba* extract, kaempferol, and silybin were prepared separately as solutions (20 mM) and extracts (10 mg/mL) and plated. Substance concentrations were converted to mg/mL equivalents to allow for comparison of botanical/dietary mixtures (with no Molar equivalent) to individual constituents of known concentration. The first two columns of each plate were empty. The plates were sealed and stored frozen until shipment for testing at the National Center for Advancing Translational Sciences (NCATS).

### Cell culture conditions

Assay descriptions and cell culture conditions have been described elsewhere^19^ and can be found in Supplementary Data.

### qHTS data analysis

The raw plate reads for each titration point were first normalized relative to the positive control compound and DMSO-only wells as follows: % Response = [(V_substance_ − V_DMSO_)/(V_pos_ − V_DMSO_)] × 100, where V_substance_ denotes the well value of a substance, V_pos_ denotes the median value of the positive control wells, and V_DMSO_ denotes the median value of the DMSO-only wells. The % Response was rescaled so that the baseline value was 0%. The dataset was then corrected by applying an NCATS in-house pattern correction algorithm.^20^ The normalized concentration–response data for each run (three runs in total) were applied to a qHTS noise filtering algorithm, CurveP, with noise level derived using both standard deviation (SD) in DMSO-only wells and the substance data from the screens (Supplementary Data).^21^ Four activity parameters, including weighted area-under-curve (wAUC, total activity), point-of-departure (POD, concentration at which the response is equivalent to the noise threshold), EC_50_ (half maximal effect concentration), and E_max_ (maximal response), were reported on each curve. The wAUC is an updated version of the original curve metric calculated by CurveP, which is now available as an R package (github.com/moggces/Rcurvep, v0.3.1).^22^ The details on the implementation can be found in the Supplementary Data. Curves with absolute wAUC >0 were considered having significant responses; curves with wAUC = 0 were considered having no significant responses. Substances with >50% of curves with significant responses (i.e., two of three concentration response curves) were assigned as active. The other activity parameters (POD, EC_50_, and E_max_) were summarized using the median value based on data from three runs (potency values were not assigned for inactive chemicals). In addition, active compounds whose effects may be associated with assay interference were labeled as inconclusive.^22^ For the real-time cytotoxicity assays, the wAUC data were integrated and the most potent POD value was used to represent the potency effect to summarize the results across multiple time points.^23^ For the AroER triscreen assay, since the effect caused by aromatase inhibition could be confounded by estrogen receptor (ER) antagonism, an additional POD comparison was done between these two effects using the same cutoff (dilution factor), and effects that did not meet the criterion were flagged.^24^ The procured sample information, concentration–response data, and data analysis results can be downloaded from the NTP Chemical Effects in Biological Systems (CEBS) database (https://doi.org/10.22427/NTP-DATA-023-00001-0001-000-7).

## Activity Data Visualization

### Heatmap & hierarchical clustering

To visualize the activity of substances (including both botanicals and their purported active constituents, *n* = 90) with regard to the 20 primary endpoints, a heatmap was plotted using log10-transformed POD values. For the activity labeled as “inactive” or “inconclusive”, a POD of 1000 μg/mL was set. The column and row arrangement on the heatmap were based on the results of hierarchical clustering (dendrograms on the heatmap). The clustering was performed using Euclidean distance for evaluating similarity between rows/columns and the average linkage criterion was used to link the groups that were similar. Annotations for the substances (types of formulation and botanical groups) and for the endpoints (effect and direction) were also added onto the heatmap. The R package *pheatmap* was used for heatmap plotting.^25^

### t-distributed stochastic neighbor embedding

The t-distributed stochastic neighbor embedding (R package, *Rtsne*) was applied to visualize the relationship between substances based on their responses in the assays.^26^ The t-SNE is a nonlinear dimensionality reduction technique for embedding high-dimensional data for visualization in a two-dimensional (2D) space. The input for the t-SNE was based on the responses (wAUC) in all assay readouts (including counter screens). The wAUC contains information illustrating both potency and efficacy across all assay readouts providing the highest granularity for differentiating botanical groups. Two datasets were used in the calculations: the first includes botanicals and their purported active constituents (# of substances = 90 and # of dimensions = 132) and the second includes marketed botanicals and Tox21 substances (# of substances = 6823 and # of dimensions = 118). Only Tox21 substances that were screened in all of the same assays as the botanicals were included in the analysis. The difference in the number of dimensions was due to the lack of some of the endpoints in Tox21 screens (e.g., no counter screens in some of the Tox21 agonist mode assays). Tox21 substances that could be autofluorescent in these assays were excluded (*n* = 227). The Euclidean distance matrix was precalculated as the input for the t-SNE. The default perplexity parameter (30) was used, except for the first dataset (25). Number of iterations was set to 5000 and principal component analysis was not used to preselect dimensions.

## Activity Enrichment Analysis

### Botanicals

To investigate which endpoint activities were enriched in certain botanical groups (19 groups), the one-way ANOVA *F*-test was applied using the log10-transformed POD data of the 20 endpoints. For the activity labeled as “inactive” or “inconclusive”, a POD of 1000 μg/mL was set. Specifically, the *F*-test statistic calculates the ratio of between-group variance to within-group variance (*F*-value). A higher *F*-value for an endpoint represents activities that were more consistent within certain botanical groups. The endpoints then were ranked by *F*-values.

### Botanicals and constituents

For the five botanical groups with both extracts and constituents, the one-way ANOVA F-test was first performed on the five groups (*F*-value 1) and then on the 11 groups, considering each type of constituent/extract as a group (*F*-value 2) based on the POD data. The ratio of *F*-value 2 to *F*-value 1 (*F*-ratio) was calculated. A higher *F*-ratio for an endpoint indicates that the activities are more consistent when considering extracts and constituents as separate groups. The endpoints thus were ranked by the *F*-ratio.

### Substance correlation analysis

To investigate sample-to-sample difference, pairwise Spearman rank correlation was calculated between botanical substances using responses (wAUC) in all assay readouts (including counter screens). For the pairs with correlation value <0, the correlation value = 0 was assigned. For the pairs in which rank correlation calculation failed (due to standard deviation = 0), a correlation value = 0 was assigned if only one substance had all zero responses, or a correlation value = 1 was assigned if both substances had all zero responses (∼1.3% of pairs).

### Substance ranking

The substances were ranked based on three scenarios: (1) number of active responses in the primary endpoints, (2) the most potent POD value from the active responses in the primary endpoints, and (3) sum of Z-score scaled wAUC values from the active responses in the primary endpoints. For the inactive or inconclusive responses, a POD of 1000 μg/mL was set for analysis. Two datasets were used separately in the Z-score scaling: the first includes marketed botanical substances and their active constituents (# of substances = 90 and # of primary endpoints = 20) and the second includes marketed botanical substances and Tox21 substances (# of substances = 6823 and # of primary endpoints = 18). The difference in the number of primary endpoints is due the use of the AroER triscreen assay in the botanical qHTS screening, but not in the Tox21 screening.

## Results

### Summary of *in vitro* cell-based assay data across all test substances

**FIG. 1.**
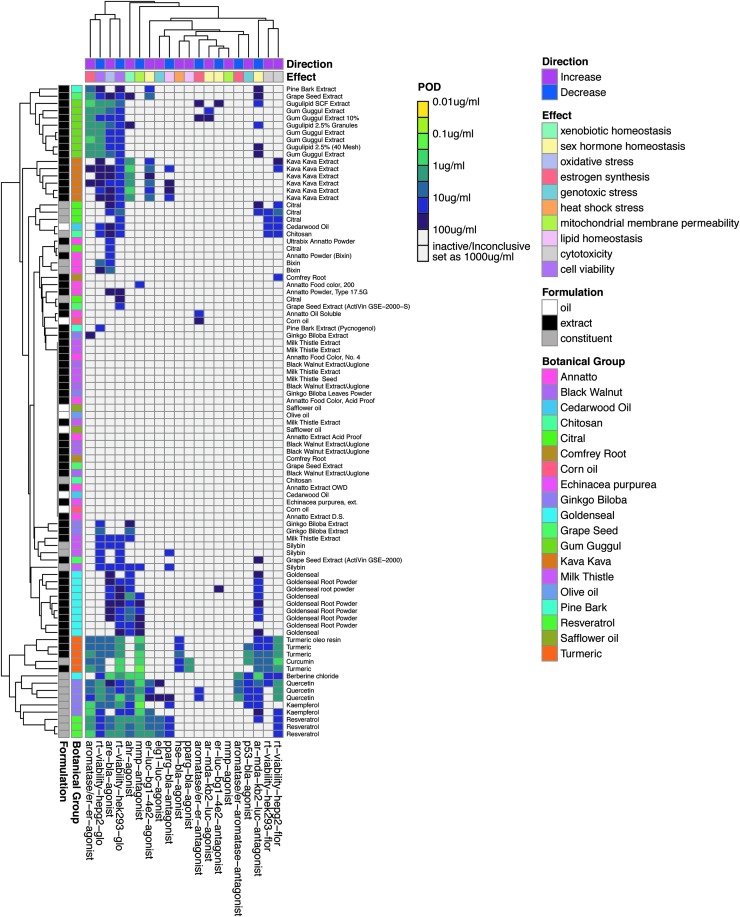
Heatmap summary of botanical/dietary substance activity in Tox21 assay panel. Each row is representative of a single test substance and each column is representative of a single *in vitro* assay. Assays are categorized by the relevant biological effect and the direction of effect being measured. The observed POD, a measure of relative potency in each assay, is indicated by the color scheme yellow (more potent) to purple (less potent). No coloration is indicative of no conclusive POD. Test substances (left; *y*-axis dendrogram) are grouped into clusters of similar activity profiles and assays are clustered (top; *x*-axis dendrogram) according to concordance of activities across test substances. POD, point of departure.

Botanical/dietary supplement biological activity was evaluated in 20 distinct assays from the Tox21 testing pipeline that encompass multiple biological responses, including cellular stress, cytotoxicity, genotoxicity, nuclear receptor activity, and cell viability. A summary of the relative activity of 90 dietary/botanical supplement lots and corresponding constituents across the 20 Tox21 assays is provided in Figure 1. Of the 19 botanical/dietary supplement groups tested, 15 had at least one lot/test material display detectable activity in at least a single assay. Promiscuous activity profiles, characterized by activity in multiple assays, and increased potency (lower POD) were evident among tested chemical constituents. Test substances with the highest relative activity included gum guggul extracts, kava kava extracts, turmeric extract, resveratrol, and marker constituents of *G. biloba* (kaempferol and quercetin), goldenseal (berberine), and turmeric (curcumin). Of the 90 test substances, 25 (27.7%) displayed no activity in tested assays. Inactive substances found in the center of the heatmap included test lots from the following botanical groups: milk thistle extract, annatto, black walnut extract, *G. biloba* leaf powder, safflower oil, olive oil, comfrey root, grape seed extract, chitosan, cedarwood oil, *Echinacea purpurea*, and corn oil.

Due to the complexity of botanical/dietary mixtures, it was unclear which *in vitro* screens would provide utility for evaluation of this class of substances. A distribution of responsivity across the testing panel was observed. Of the 20 assays selected for this study, evaluations of stress response (decreased mitochondrial membrane potential [MMP] and increase in nuclear factor E2-related factor 2 [Nrf2] transcriptional activity), measurement of decreased cell viability by RealTime-Glo (in both HepG2 and HEK293 cell), and select nuclear receptor activities (increased aryl hydrocarbon receptor [AHR]/ER transcriptional activity, in MCF7 cells) were the most responsive endpoints across the test substances. In contrast, assays designed to measure decreased ER transcriptional activity (in both BG1 and MCF-7 cell lines), increased androgen receptor (AR) transcriptional activity, and increased MMP were the least responsive endpoints across tested botanicals.

### Comparison of activity profiles of dietary/botanical supplements

**FIG. 2.**
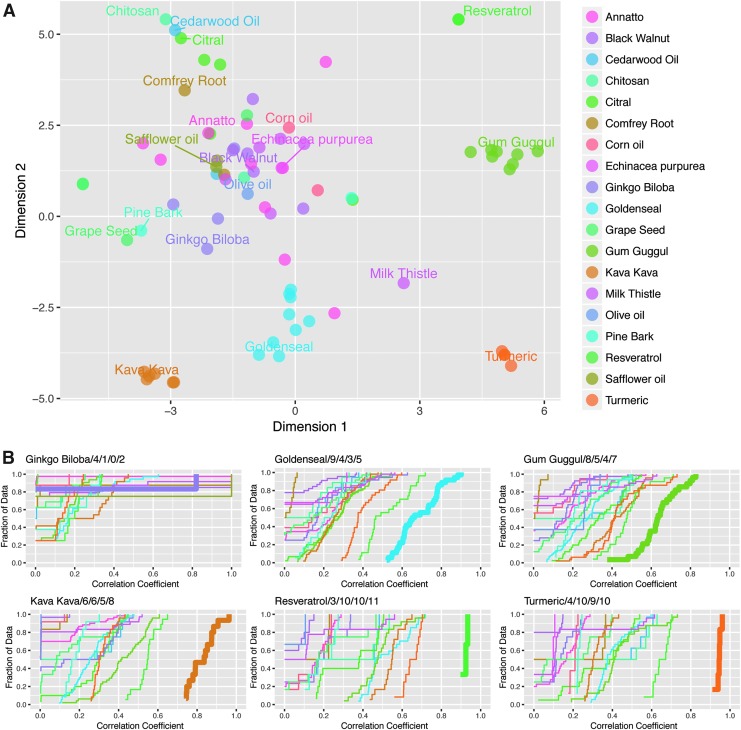
Comparison of botanical/dietary supplement *in vitro* activity profiles. **(A)** tSNE clustering summary based on the responses (wAUC) in all assay readouts for botanical test substances, excluding individual constituents. **(B)** Comparison of botanical/dietary supplements (those with ≥3 different test lots) by pairwise Spearman rank correlations using responses (wAUC) in all assay readouts. Plots are labeled by botanical group (*Ginkgo biloba*, goldenseal, gum guggul, kava kava, resveratrol, and turmeric) followed by four numbers, indicating the number of lots assessed/average number of active assays across lots/minimum activity by a single lot/maximum activity by a single lot. The distribution of the thick line across the *x*-axis represents the degree of similarity within a group (i.e., steeper line corresponds to higher degree of similarity). The degree of separation between the thick line and the thin lines represents the uniqueness of the group relative to other tested botanical/dietary supplements (i.e., greater separation corresponds to a more unique activity profile of the botanical compared to other tested botanicals). tSNE, t-distributed stochastic neighbor embedding; wAUC, weighted area under curve.

Initial comparisons of activity were restricted to dietary/botanical supplement extracts. A tSNE dimension reduction method was used to compare and visualize botanical/dietary supplement extracts across the spectrum of measured endpoints (Fig. 2A). These data display distinct clustering profiles among the tested formulations, with data points in closer proximity representing similar qHTS activity profiles. In many cases, different lots of a single botanical/dietary supplement form a distinct cluster, such as gum guggul, goldenseal, turmeric, and kava kava extracts. These clusters indicate unique activity profiles associated to a specific botanical ingredient and limited variability among tested lots. In contrast, a number of botanical groups exhibited an overall lack of measurable activity across the currently assessed bioactivity space. Milk thistle presented a unique case in which a single test lot displayed an activity profile that was dissimilar from other milk thistle lots. While four milk thistle extract and one milk thistle seed samples were inactive, one milk thistle extract sample displayed measurable activity in 4 of the 20 test assays.

To further compare variations in activity between samples, pairwise Spearman rank correlations were calculated between botanical/dietary substances with ≥3 test samples using responses (wAUC) in all assay readouts. For each of the 11 botanical/dietary supplement groups with ≥3 test samples, cumulative distribution plots were generated to assess within-group (thick line) and between-group (thin line) correlations (Fig. 2B). The distribution of the thick line across the *x*-axis represents the degree of similarity within a distinct botanical/dietary supplement group (i.e., the steeper the line, the more similar group members are to one another). The thin lines represent the activity profile of the most active lot within a botanical group. Botanical groups that produced no measurable activity could not be included in this analysis. The degree of overlap between the thick line and the thin lines represents the uniqueness of the group compared with other tested botanical/dietary supplements. For example, the turmeric group contains four samples and on average is active in 10 assays (out of 20). The within-group correlation (thick line) is over 0.9 on the *x*-axis, indicating the activities presented in this group are very consistent across tested lots. The lack of overlap with other botanical groups (thin lines) indicates that observed activities are unique to turmeric relative to other assessed botanical groups. In contrast, *G. biloba* contains four samples within the group, but on average, there is only a single active response for each sample. For five out of six (0.83) pairs, the correlation = 0, but there is only a single pair showing a correlation of 0.8. Resveratrol and turmeric show significant within-group similarity and between-group uniqueness. The within-group similarity for kava kava, gum guggul, and goldenseal was not as consistent as the resveratrol and turmeric examples, but they are also unique relative to other botanical groups. The remaining botanical groups could not be differentiated from the other groups due to general inactivity in the test panel or highly variable within-group activity, as is the case with milk thistle (Supplementary Fig. S1).

**FIG. 3.**
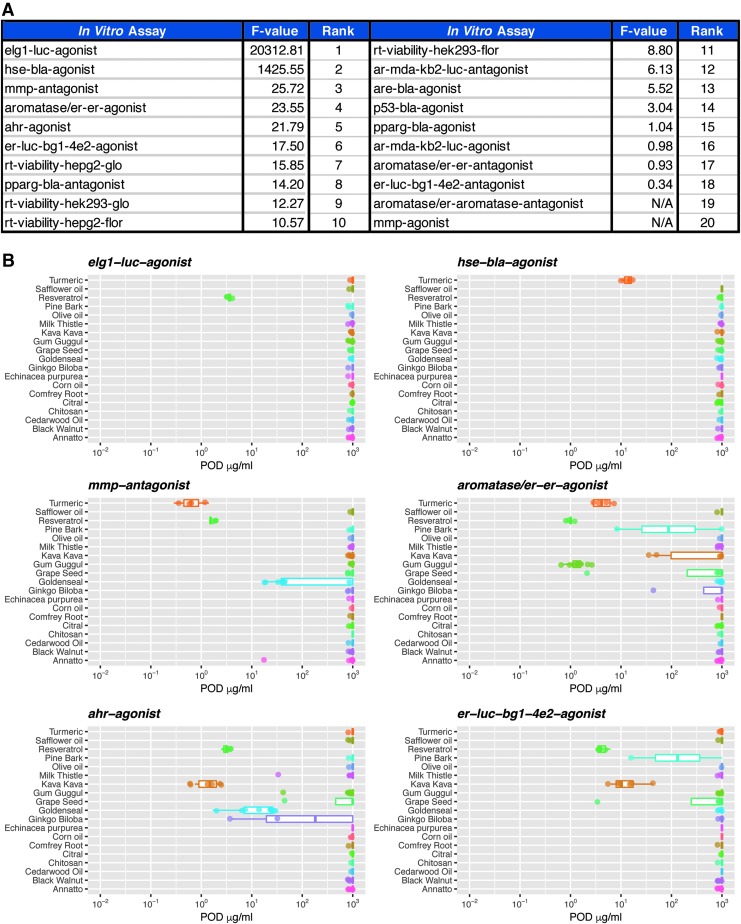
Comparison of differentiation activity among botanical/dietary supplements. **(A)** Assays were ranked by corresponding *F*-value, with higher *F*-values associated with endpoints that were more consistent within certain botanical groups and contributed more to variability in activity profiles between tested botanical/dietary supplements. **(B)** Box plots summarizing relative POD data in the top six ranked assays (based on *F*-value) across tested botanical groups.

*In vitro* assays that produced differential responses across test agents help identify biological variability. *F*-values were calculated based on the ratio of between-group variance to within-group variance for each of the 20 primary endpoints to distinguish which assays are the primary determinants for differential activity among tested botanical/dietary supplements, (Fig. 3A). Higher *F*-values indicate an increased confidence that a specific assay contributed to the unique biological activity profile of a botanical group. The potency results of the top six endpoints from the F-statistic are presented in Figure 3B and include increased ELG1 transcriptional activity, increased heat-shock factor transcriptional activity, decreased MMP, increased ER transcriptional activity in MCF7 cells, and increased AHR transcriptional activity. From the box and whisker plots, it can be seen that botanicals that formed distinct clusters in Figure 2A (resveratrol, turmeric, kava kava, gum guggul, and goldenseal) were more potent in these assays relative to other test substances. Potency plots for the assays with lower *F*-values can be found in Supplementary Figure S2.

**FIG. 4.**
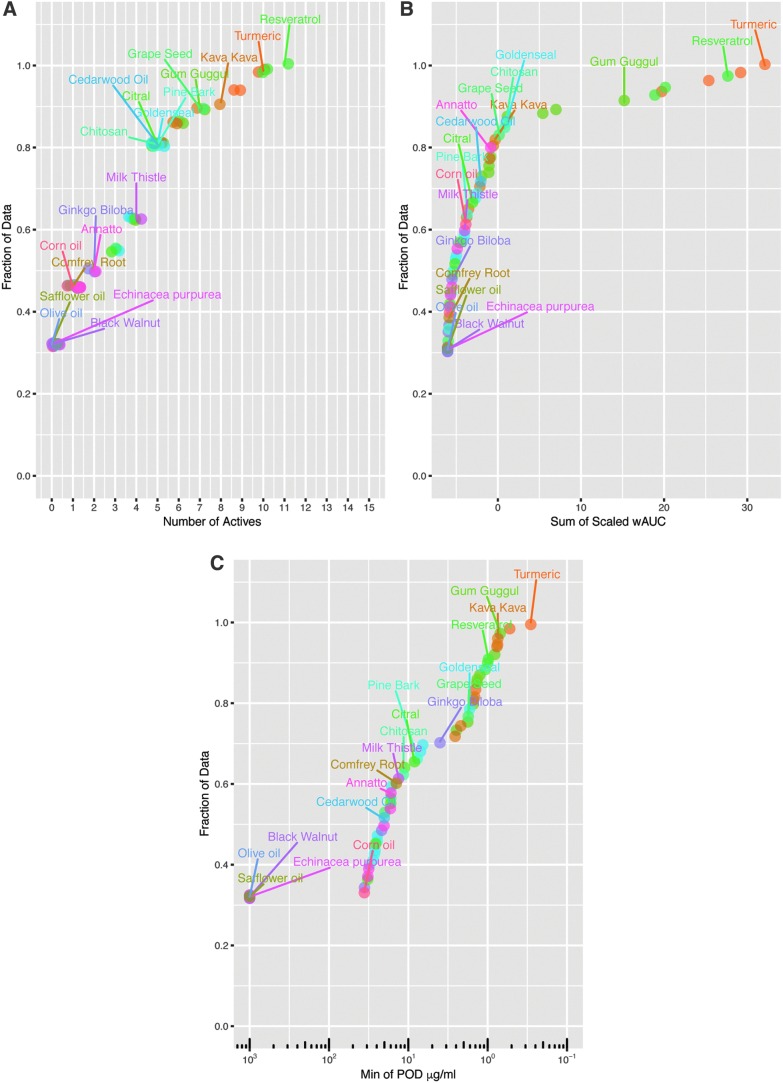
Comparison of cumulative biological activity across tested botanical/dietary supplements. The most active test lot within a botanical group is labeled. Cumulative distribution plots of botanical/dietary extract biological activity rank based on **(A)** number of assays in which the test substance was active, **(B)** sum of the scaled wAUC across all assays, and **(C)** minimum POD observed in the testing panel.

Knowledge of specific molecular targets can be useful for mechanistic understanding; however integration of multiple *in vitro* endpoints to gain a sense of overall biological activity can be informative for prioritization approaches.^17^ Overall biological activity of botanical/dietary supplements was evaluated using two different ranking criteria: number of active assays and sum of the scaled wAUC. The results are presented as cumulative distribution plots with the most active test lot within a botanical group labeled. The number of actives classification criteria characterizes broad biological activity and is not heavily weighted by increased efficacy/potency in a singular assay (Fig. 4A). About 80% (63/78) of test substances are active in fewer than five assays. Resveratrol, turmeric, and kava kava displayed the largest range of activity in 11, 10, and 8 separate assays, respectively. Using the wAUC criteria, the summation of activity across all assays is totaled, such that potency and efficacy in individual assays do not contribute equally to the overall biological activity ranking (Fig. 4B). Botanical/dietary supplements that displayed the largest summation of biological activity included turmeric, resveratrol, gum guggul, and kava kava, respectively. In addition, botanical/dietary test substances were ranked by the minimum POD observed across all assays, with the highest *in vitro* potency associated with turmeric, gum guggul, kava kava, and resveratrol, respectively (Fig. 4C). While the same botanical groups are continually observed as highly active, their relative ranking of activity is altered by the applied selection criteria.

### *In vitro* activity comparison of botanical supplements versus marker constituents

**FIG. 5.**
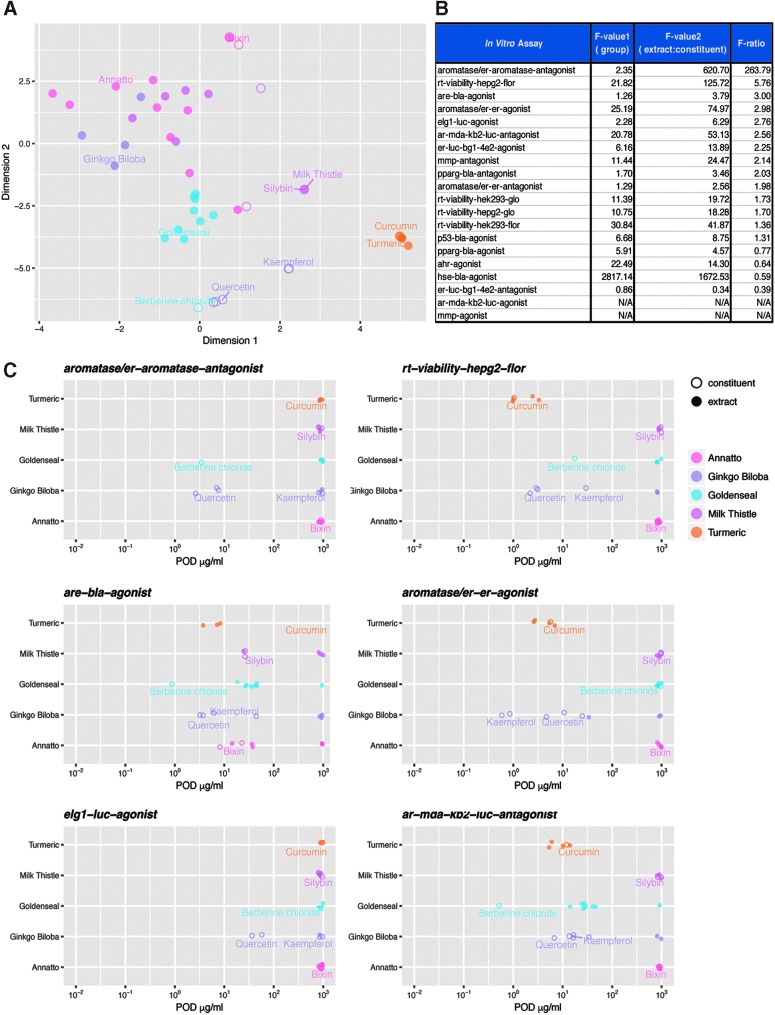

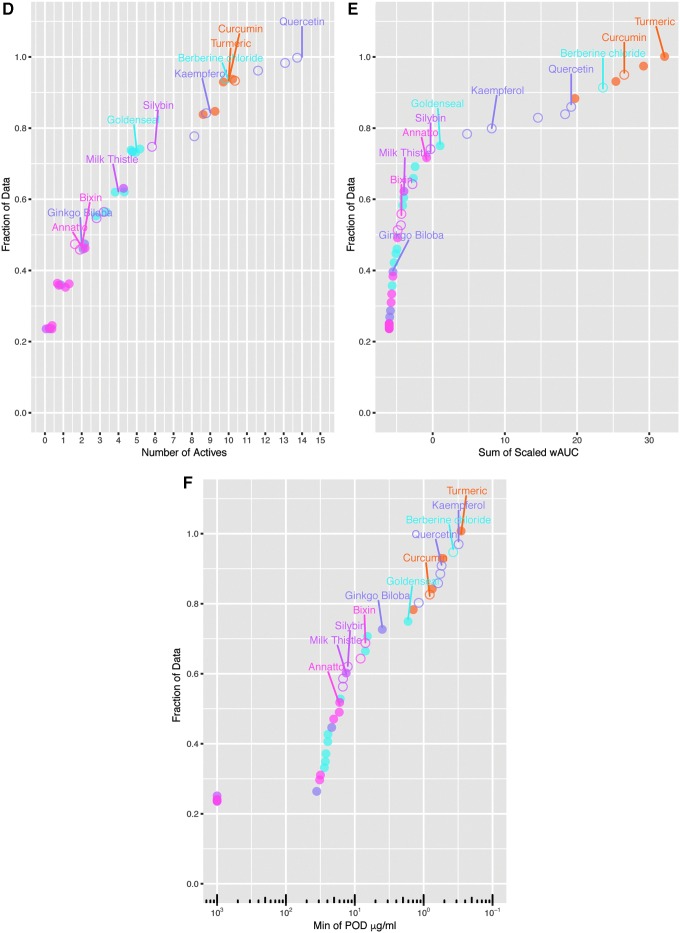
(Continued).

The determinant of biological activity for complex botanical mixtures can be viewed as a dichotomy with two schools of thought. The first being that observed biological activity is a result of the integrated activity of numerous chemical constituents. The other being that activity/toxicity of a botanical supplement is primarily due to the presence of an individual constituent. Therefore, we sought to compare the biological activity profile of botanical supplements relative to known marker constituents. A tSNE dimension reduction method was used to compare and visualize the activity profile of botanical supplement extracts relative to marker constituents across the spectrum of measured endpoints (Fig. 5A). The cluster analyses indicate that turmeric and its marker constituent curcumin produce similar activity profiles. In contrast, *G. biloba* and goldenseal do not cluster with their respective marker constituents, quercetin/kaempferol and berberine, respectively. Annatto and milk thistle display significant variations among botanical test lots and corresponding marker constituents, bixin and silybin, respectively. Both annatto and milk thistle present a case in which an individual constituent clusters with a single lot of their respective botanical. However, due to the variable profiles of individual chemical constituents and lack of quantitative chemistry data, these results were difficult to interpret.

*F*-ratio calculations were used to rank biological assays that displayed the highest variability between a botanical and its corresponding marker constituent(s) (Fig. 5B). The potency results of the top six endpoints based on *F*-ratio values include aromatase inhibition, cytotoxicity in HepG2 cells, increased Nrf2 transcriptional activity, increased ER transcriptional activity in MCF7 cells, increased ELG1 transcriptional activity, and decreased AR transcriptional activity (Fig. 5C). Potency plots for assays that correspond to the 12 lowest *F*-ratio values can be found in Supplementary Figure S3. These rankings were primarily driven by differences in activity between goldenseal extracts and berberine, as well as *G. biloba* extracts and quercetin/kaempferol. Turmeric and its constituent curcumin were both similarly active across the test panel. Extracts and constituents of annatto and milk thistle were mostly inactive and did not display distinguishable differences in activity. Overall biological activity of botanical/dietary supplements relative to their constituents was evaluated using two different ranking criteria: number of active assays and sum of the scaled wAUC. The results are presented as cumulative distribution plots with the most active test lot within a botanical group labeled (Fig. 5D, E). With the exception of turmeric, individual constituents (curcumin, berberine, quercetin, and kaempferol) were more active than botanical extracts with activity in at least 8 of the 20 tested assays. Quercetin displayed the widest breath of biological activity of all test substances, with marked activity in 14/20 (70%) of the tested assays. Turmeric, curcumin, berberine, quercetin, and kaempferol displayed the largest summations of overall biological activity, respectively. Turmeric was the only botanical extract that mediated a minimum POD that was less than a 1 μg/mL extract equivalent (Fig. 5F). Kaempferol, berberine, quercetin, and curcumin also displayed a minimum POD less than a 1 μg/mL extract equivalent.

### Botanical/dietary extract activity versus Tox21 10k library

**FIG. 6.**
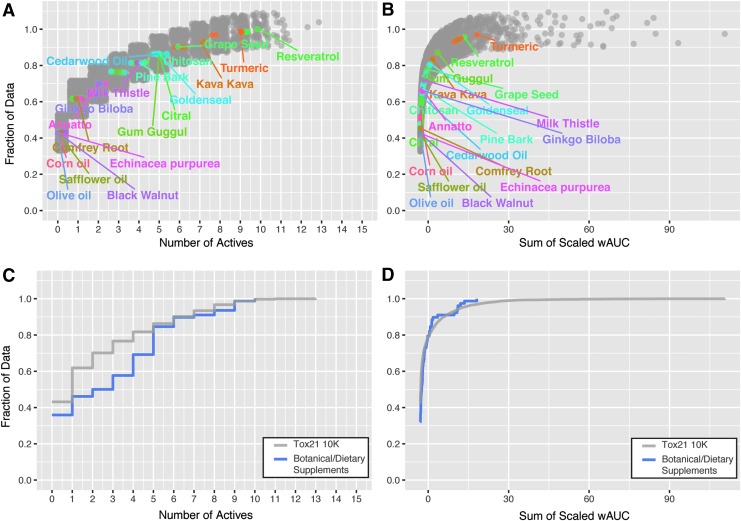
Relative activity of botanical/dietary extracts to the Tox21 10k library. Chemical library data were restricted to 6745 chemicals. The most active test lot within each botanical group is labeled. Cumulative distribution plot of botanical/dietary extract and Tox21 library biological activity rank based on **(A)** number of assays in which the test substance was active and **(B)** sum of the scaled wAUC across all assays. Cumulative line plots indicate fraction of Tox21 (gray line) and botanical/dietary extracts (blue line) that correspond to cumulative activity based on **(C)** number of actives and **(D)** sum of scaled wAUC data.

Relative to each other, botanical/dietary supplements display a wide range of measurable activities. However, it is not known how such activities compare to the entirety of the chemical space previously analyzed in the Tox21 10k library. The library is a compendium of greater than 8000 unique compounds, containing industrial chemicals, flame retardants, pesticides, plasticizers, food additives, therapeutic agents, and chemical synthesis byproducts.^16^ Due to identified differences in assay methods, the chemical library evaluated herein was limited to 6745 chemicals. Botanical/dietary test substances clustered among Tox21 samples in a t-SNE dimension reduction analysis, indicating similar measured biological activity (Supplementary Fig. S4). A large portion of the Tox21 library clustered together due to complete inactivity in the utilized *in vitro* test panel. Overall biological activity of botanical/dietary supplements and the Tox21 10k library were compared using number of active assays, and sum of the scaled wAUC ranking criteria. The results are presented as cumulative distribution plots with the most active test lot within a botanical group labeled (Fig. 6A, B). Botanical supplement activities were consistent with the spectrum of activity observed in the Tox21 10K chemicals. Approximately 35% of the 78 assessed botanical/dietary supplements were inactive in all tested *in vitro* assays, relative to ∼43% of the Tox21 library (Fig. 6C). The Tox21 10K library has a higher proportion of chemicals (∼77%) that are active in four assays or less, relative to 58% of tested botanical/dietary supplements. Ninety percent of 10K library chemicals and botanical/dietary supplements were active in seven assays or less. Overall activity across all assays (sum of scaled wAUC) was comparable between 10K library chemicals and botanical/dietary supplements (Fig. 6D). Only 3.1% of the assessed Tox21 chemical library had a higher sum of scaled wAUC than turmeric (the botanical extract with the highest sum wAUC).

## Discussion

In alignment with NTP's Dietary Supplements and Herbal Medicines Initiative and in support of agency goals to develop, establish confidence in, and ensure use of new alternative testing approaches, a proof-of-concept study was conducted to evaluate the utility of assessing botanical/dietary supplements using the Tox21 *in vitro* screening panel. From this study, we find that botanical dietary supplement extracts induce measurable and diverse activity in an array of *in vitro* assays. Moreover, the distribution of bioactivity was comparable to distributions exhibited by compounds across the Tox21 10K library. As a group, botanical supplements do not exhibit minimal or idiosyncratic activities that would preclude the use of qHTS platforms as a feasible method to screen the biological activity of this class of compounds. While there was bioactivity information to be gained from qHTS of botanical dietary supplements, there exist numerous challenges and considerations when attempting to use such methods for prioritization or safety assessment purposes.

Due to their inherent complexity and variability, botanical dietary supplements continue to present many unique challenges to safety characterization by *in vitro* test systems. One limitation of this study is the lack of chemical characterization of the tested botanical extracts. Quantitation of constituent levels can provide important context for interpreting the differences in overall biological activity between different botanical lots and types of botanical extracts with their respective marker constituents. In this study, the top six assays that accounted for variances in bioactivity between tested extracts and constituents (aromatase inhibition, HepG2 cell cytotoxicity, increased Nrf2 transcriptional activity, increased ER transcriptional activity in MCF7 cells, increased ELG1 transcriptional activity, and decreased AR transcriptional activity) were largely driven by assays in which marker constituents were found to be highly active and corresponding extracts displayed no activity. It is not known whether the difference in assay-specific activity or overall lack of activity in botanical extracts is due to limited concentration of the marker constituents or a whole mixture effect (e.g., antagonistic interactions among constituents).

Constituent-level characterizations using chemometric and *in silico* modeling approaches have been used to identify potential hazards and establish safe levels of botanical use for humans.^27,28^ This method relies upon a multidetector approach to first comprehensively assess constituent levels in botanical preparations. This is followed by an *in silico* decision-tree approach that includes the following: quantitative benchmarking of components to similar compounds commonly found in foods or botanicals with well-established safety profiles, systematic evaluation of toxicity data based on structure-activity relationships, and comparisons to established thresholds of toxicological concern in absence of safety data or structural analogs.^28^ While these approaches have had success in characterizing potential hazard of botanical supplements and identifying gaps in safety data for specific constituents of concern, they rely on the *a priori* assumption that bioactivity is a consequence of the independent activity of individual chemical constituents present in botanical mixtures. However, there are numerous indications that the overall biological activity of botanical dietary supplements is determined by the concomitant activity of multiple constituents. Cotreatment with different ginsenosides (derived from *Pananx ginseng*) was able to synergistically induce antioxidant activity as measured by nuclear factor (erythroid-derived 2)-like 2 (Nrf2) transcriptional activation in human Hep-G2-C8 cells.^29^ In a study of the anticancer effects of green tea by Fu et al., decreased pulmonary adenoma formation in A/J mice was associated with exposure to polyphenon-E mixtures that contained epigallocatechin 3-gallate.^30,31^ *In vitro* or *in vivo* approaches that allow integration of the milieu of molecular signals that ultimately dictate an observed biological response are critical for evaluating mixture effects that can be greater or lesser than the additive response of components assessed individually.

One goal of the Tox21 testing program was to increase the efficiency of hazard characterization. However, numerous technical and biological modeling limitations hinder the toxicological utility for safety/hazard classifications of botanical/dietary supplements. The capacity to mimic human exposure presents a significant challenge when utilizing *in vitro* assay systems that must adhere to technical aspects required for inclusion in the Tox21 testing program. In many cases, human exposures to botanical dietary supplements occur over significant periods of time. Currently, Tox21 qHTS assays are limited to acute exposure scenarios that last no more than 48 hours.^32^ Current methods require test articles to be dissolved in DMSO, which may not accurately reflect the constituent profile resulting from ingestion of a botanical dietary supplement. Consequently, solvent selection could impact observed biological activity as has been demonstrated with the North American ginseng root (*Panax quinquefolius*), in which immune-stimulation or immune-modulatory effects are associated with aqueous and ethanolic preparations, respectively.^33–35^ Biologically or toxicologically active constituents of botanical dietary supplements may require metabolic activation by endogenous enzymes to propagate an effect. For example, cytotoxic/genotoxic and chemopreventative activities of catechol-containing natural products are likely associated with their metabolism to reactive *o*-quinones.^36^ The Tox21 assay panel utilizes 2D-cell culture models and immortal cell lines that possess limited biotransformative capacity relative to primary human hepatocytes.^37^ HepaRG three-dimensional spheroid models are a promising alternative for use in the Tox21 testing panel due to their metabolic competence and expression of physiologically relevant levels of critical xenobiotic metabolism enzymes.^38^

Comparisons of relative potencies of botanical extracts to individual chemicals present in Tox21 represent an additional challenge. Equimolar preparations of individual chemicals can be compared such that the molecular weight of a chemical does not confound estimates of relative potency. Due to the complex composition of botanical/dietary supplements, exposure levels are often expressed in terms of mass-extract equivalents. For comparison purposes, both botanical extracts and individual chemical preparations can be converted to mass-extract equivalents resembling relative potencies based on a mg/kg dosage scale. In addition, relative activities and potency estimates are based on applied nominal exposure concentrations, when actual exposure of cell systems may not equate to the levels presumed in cell culture media, further confounding concentration–response interpretation.

Currently, the Tox21 and EPA ToxCast programs include over 800 distinct measurable endpoints. Future use of qHTS platforms to evaluate or predict botanical safety will likely require identification of a more manageable *in vitro* testing panel. In this study, select assays were of increased utility in the characterization of differential activity between tested botanical groups (increased ELG1 transcriptional activity, increased heat-shock factor transcriptional activity, disruption of MMP, increased of ER transcriptional activity in both BG1/MCF7 cells, and increased AHR transcriptional activity). While these assays were informative, relative to those that showed little measurable activity (MMP-agonist and ER-antagonist assays), they provide a limited range of assessed biological-response coverage necessary for identification of hazardous botanical agents. Both comfrey root and *G. biloba* extracts displayed little biological activity in the testing panel, which could be interpreted as inertness, and suggest limited hazard potential. Conversely, there are concerns regarding consumption of comfrey root and *G. biloba* due to observed toxic and carcinogenic activities.^39–41^ It is important to note that assay utility is highly dependent upon the chemical profile of the tested herbal and the molecular mechanisms by which specific constituents influence biological activity or cause toxicity. Therefore, *in vitro* assay panels are unlikely to be a “one size fits all” testing paradigm, but will need to be fit for purpose to adequately characterize the bioactivity of diverse botanical test substances. Currently, the Tox21 testing pipeline is highly efficient for generating “biological fingerprint” profiles for a large library of chemical test substances. However, the current testing framework is resource intensive and may not be amenable to implementation in other research settings. To ensure identification of hazardous botanical substances using *in vitro* systems, further endpoints or modifications to the testing framework could be incorporated, which capture additional pathways/mechanisms with toxicological relevance. In cases when a putative therapeutic mode of action has been identified, it may be of value to include assays that evaluate such activities. For example, the pharmacological effects of valerian extract and valerenic acid are thought to be mediated through modulation of gamma-aminobutyric acid receptor function.^42^ Assays evaluating neuronal cell firing using multielectrode arrays may be useful in evaluating lots of valerian root extract. Furthermore, institution of a testing framework that utilizes chemometric profiling coupled with QSAR modeling, similar to Little et al., could provide a mechanism to prioritize and predict assay panels that will provide relevant toxicological and biological activity information based on the chemical profile of the botanical test substance.^28^ Tox21 phase III focuses on alternative methods (e.g., transcriptomics) that represent a high content alternative to the limited pathway readouts assessed in the current testing panel. Transcriptomic methods such as the S1500+ gene set provide a more comprehensive assessment of the biological system by assessing perturbations in the expression of over 1500 genes.^43^ Tox21 Phase III methods may be more easily adapted for implementation in academic research settings than the current Tox21 phase II testing framework. However, further validation would be necessary for integrative use of transcriptomic approaches in hazard and safety assessments of botanical/dietary supplements.

Interpretation of observed biological activity of botanical dietary supplements can be further confounded by the presence of Pan Assay Interference Compounds (PAINS) that are common to many natural products.^44^ PAINS have specific structural motifs that interfere with *in vitro* cell-based assays through various mechanisms such as reactivity, redox cycling, membrane disruption, metal chelation, and colloidal aggregation.^45^ Therefore, PAINS tend to be promiscuously active across numerous bioassays. Major resources have been expended to investigate the pharmacological potential of these presumed active substances.^46^ Although the nonspecific activity of PAINS can severely confound interpretation of *in vitro* assay data, it should not be inferred that these substances are biologically inert or would not produce *in vivo* effects. Of the botanical constituents investigated in this study, quercetin, kaempferol, and curcumin are well-known PAINS and contain structural motifs with high promiscuity.^47^ Notably, these compounds were among the most biologically active observed in this study. Berberine did not show high bioassay promiscuity, suggesting that the activities identified in this study may be more relevant/accurate for berberine than for quercetin, kaempferol, or curcumin.

Going forward, there are many examples in which *in vitro* testing of botanical dietary supplements can be of great utility. Bioassay-guided fractionation techniques that combine analytical separation of botanical mixtures and *in vitro* or *in vivo* assay activity, can be used to identify a previously unknown biologically active or toxic constituents.^48^ Use of this methodology led to the identification of the active constituent of locoweed (Genera: *Oxytropis* and *Astragalus*), which caused numerous incidences of livestock poisoning in the Western United States.^49,50^ By first identifying α-mannosidase inhibition as the mechanism of locoweed toxicity, fractionations of locoweed could be assessed for this specific activity, which ultimately was linked to the constituent swainsosine.^50^ *In vitro* assessments of botanical dietary supplements have been utilized by the NTP in an effort to develop methods to assess “sufficient similarity” of complex mixtures. A recent case study of *G. biloba* extracts found that by using targeted chemistry, untargeted chemistry, *in vitro* liver models, and short-term animal models, different test lots could be deemed similar or different to a reference sample.^51^ By demonstrating sufficient similarity, it is possible to infer that a specific botanical supplement or mixture will display a similar toxicological profile to the previously tested sample, eliminating the need for additional *in vivo* studies.

One of the greatest concerns regarding the use of botanical dietary supplements is the potential for botanical-drug interactions. Use of botanical supplements among adult individuals is correlated with being uninsured and increased use of over-the-counter or prescription medications.^52,53^ Many botanical species are known to influence the activity of various drug metabolism pathways, ultimately mediating the increased or decreased pharmacologic activity of various medications. *E. purpurea* root modulates the activity of cytochrome P450 (1A2/3A), which can thereby influence the effective dose of drugs that are activated or metabolized by this mechanism.^54^ *In vitro* assays can provide an efficient means to screen for potential modulatory effects of botanical dietary supplements on critical drug metabolism pathways.^55^

In summary, this case study illustrates that the Tox21 testing approach is a useful tool to assess numerous facets of biological activity of botanical dietary supplements. However, there are still many considerations when attempting to use *in vitro* qHTS methods to characterize the safety profile of test substances. Further development is required to facilitate integration and interpretation of these complex data to better understand how results compare to traditional methods and how these data can be translated to assist in regulatory decision-making.

## Supplementary Material

Supplemental data
