# Supplementary material for: Using Tox21 High-Throughput Screening Assays for the Evaluation of Botanical and Dietary Supplements
Source: Appl In Vitro Toxicol. 2019 Mar 9;5(1):10–25. doi: 10.1089/aivt.2018.0020 (PMC6442399; doi:10.1089/aivt.2018.0020)
Supplement: Supplemental data [file Supp_Data.pdf]

Supplemental Materials

Supplemental Methods

Test Substance Preparation and Dosing: For two test substances (kaempferol and silybin), the solution and extract were prepared from the same lot of material. Two constituent solutions (citral and curcumin) were plated in duplicate to fill a total of 352 wells. For this study, the log10 of dilution factor (square root of 5) was used.

Cell Culture Conditions: HepG2 and HEK-293 cells were purchased from American Type Culture Collection (ATCC, Manassas, VA). HepG2 cells were cultured in Eagle's Minimum Essential Medium (EMEM, ATCC) and HEK-293 cells in Dulbecco's Modified Eagle's Medium (DMEM, Life Technologies Corporation, Carlsbad, CA), and both supplemented with 10% Hyclone's Fetal Bovine Serum (FBS; ThermoFisher Scientific, Waltham, MA), and 100 U/ml penicillin-100 µg/ml streptomycin (Life Technologies Corporation).

MDA-kb2-AR cell line was purchased from ATCC, and was developed from the parental cell line MDA-MB-453 of human breast cancer cells, expresses firefly luciferase under control of the MMTV promoter that contains response elements for both glucocorticoid receptors (GR) and androgen receptors (AR). MDA-kb2-AR cells were cultured in L-15 Medium (ATCC) supplemented with 10% FBS, and 100 U/mL penicillin-100 µg/mL streptomycin.

ATAD5 cell line was provided by Dr. Kyungjae Myung (NHGRI, NIH) and contains a firefly luciferase reporter-gene tagged with ATAD5.[1] ATAD5 cell line was used identify the compounds that cause DNA damage/genetic stress. ATAD5 cells were cultured in DMEM supplemented with 10% FBS, and 100 U/ml penicillin-100 µg/ml streptomycin.

MCF-7 aro-ERE cell line provided by Dr. Shiuan Chen (Beckman Research Institute, CA) is a human breast carcinoma cell line that was stably transfected with a promoter plasmid, pGL3-Luc, containing three repeats of estrogen responsive element (ERE). MCF-7 aro-ERE cells were used to screen aromatase inhibitors, estrogen receptor (ER) agonists and ER antagonists.[2] MCF-7 aro ERE cells were cultured in MEM/EBSS Medium (Hyclone) supplemented with 10% FBS, 20 μg/ml Hygromycin, 50 μg/ml G418 and 100 U/mL penicillin-100 μg/mL streptomycin.

VM7Luc4E2 and HG2L7.5c1 cell lines were provided by Dr. Michael S. Denison (University of California, Davis). VM7Luc4E2 endogenously expresses full-length ERα and is stably transfected with a plasmid containing four estrogen responsive elements (ERE) upstream of a luciferase reporter gene. VM7Luc4E2 cells were used to screen ERα agonists and antagonists. Whereas HG2L7.5c1 cells were used to identify the compounds that induce AhR activation and contains HepG2 cells that are stably transfected with an Aryl hydrocarbon Receptor (AhR)-responsive firefly luciferase reporter. Both VM7Luc4E2 and HG2L7.5c1 cells were cultured in MEMα medium (Life Technologies) supplemented with 10% Premium FBS (Atlanta Biologicals, Flowery Branch, GA), 400 μg/ml G418, and 100 U/mL penicillin-100 μg/mL streptomycin.

HSE-*bla* Hela, ARE-*bla* HepG2, PPAR gamma-UAS-*bla* 293H, and p53-*bla* HCT-116 cells were purchased from Life Technologies. These cell lines contains a β-lactamase reporter gene under control of a heat shock response element (HSE), antioxidant response element (ARE), peroxisome proliferator-activated receptor gamma (PPAR-gamma), and p53 response elements that have been stably integrated into HeLa, HepG2, 293 H, HCT-116 cells respectively. All the recommended culture medium components were purchased from Life Technologies. All cells were cultured and maintained at 37°C under a humidified atmosphere and 5% CO_2, except MDA-kb2-AR cells which were cultured and maintained at 37°C under a humidified atmosphere and 0% CO_2. All detailed

descriptions of the assays are publicly available through the NCATS website (https://tripod.nih.gov/tox21/assays/) and PubChem.

ATAD5, AR-MDA, and AhR luciferase (luc) reporter gene assays: ATAD5-Luc, MDA-kb2-AR, and HG2L7.5c1 cells were dispensed at 2000, 3000, and 4000/5 µL/well respectively and 3000/4 µL/well (MDA-kb2-AR antagonist mode) in 1536-well tissue culture treated white wall/solid bottom plates (Greiner Bio-One North America, Monroe, NC) using a Multidrop Combi dispenser (ThermoFisher Scientific, Waltham, MA). After the assay plates were incubated at 37°C for 5 h, 23 nL of compounds dissolved in DMSO, positive and negative controls were transferred to the assay plates using a Pintool station (Wako, San Diego, CA). For MDA-kb2-AR antagonist mode, 1 µL/well agonist (10 nM R1881) or assay medium was added to each well using a Flying Reagent Dispenser (FRD, Aurora Discovery, Carlsbad, CA). The assay plates were incubated for 16 h (ATAD5-Luc and MDA-kb2-AR) and 24 h (HG2L7.5c1) at 37°C. For cell viability assay, 1 µL/well CellTiter-Fluor™ Cell Viabillity reagent (Promega Corporation, Madison, WI) was added to each well using an FRD. After 0.5 h incubation at 37°C, the fluorescence intensity was measured using a ViewLux plate reader (PerkinElmer, Shelton, CT). For luciferase reporter gene assay, 4 µL/well Amplite™ Luciferase Assay reagent (ATAD5; AAT Bioquest®, Inc. Sunnyvale, CA) and ONE-Glo™ Luciferase Assay reagent (MDA-kb2-AR and HG2L7.5c1; Promega Corporation) was added to each well using an FRD. After 0.5 h incubation at room temperature, the luminescence intensity was measured using a ViewLux plate reader. Data were represented as relative fluorescence units (cell viability assay) and relative luminescence units (luciferase reporter assay).

MCF-7 aro-ERE and ER-vMCF-7-Luc luciferase reporter gene assays: MCF-7 aro-ERE and VM7Luc4E2 cells were dispensed at 1500 and 4000 respectively in 5 µL/well (agonist mode) and

4 μL/well (antagonist mode) in 1536-well tissue culture treated white wall/solid bottom plates (Greiner Bio-One) using a Multidrop Combi dispenser. After the assay plates were incubated at 37°C for 5 h (MCF-7 aro-ERE) and 24 h (VM7Luc4E2), 23 nL of compounds dissolved in DMSO, positive and negative controls were transferred to the assay plates using a Pintool station. For antagonist modes, 1 μL/well agonist (0.2 and 0.5 nM β-Estradiol for MCF-7 aro-ERE and VM7Luc4E2 respectively and 0.5 nM Testosterone for MCF-7 aro-ERE testing aromatase activity) or assay medium was added to each well using an FRD. The assay plates were incubated for 22 h (VM7Luc4E2) and 24 h (MCF-7 aro-ERE) at 37°C. For cell viability assay, 1 μL/well CellTiter-Fluor™ Cell Viabillity reagent (Promega Corporation, Madison, WI) was added to each well using an FRD. After 0.5 h incubation at 37°C, the fluorescence intensity was measured using a ViewLux plate reader. For luciferase reporter gene assay, 4 μL/well ONE-Glo™ Luciferase Assay reagent was added to each well using an FRD. After 0.5 h incubation at room temperature, the luminescence intensity was measured using a ViewLux plate reader. Data were represented as relative fluorescence units (cell viability assay) and relative luminescence units (luciferase reporter assay).

Real Time Cell Viability Assay: To monitor cytotoxicity and cell viability in real time of continues cell culture, a multiplex assay by combining RealTime-Glo™ MT Cell Viability Assay (Promega Corporation) and CellTox™ Green Cytotoxicity Assay were used.[3] The mixture of HepG2 cell or HEK293 cell suspension and reagents were dispensed at 600 cells/6 μl/well in 1536-well tissue culture treated black wall/solid bottom plates (Greiner Bio-One) using a Multidrop Combi dispenser. After the assay plates were incubated at 37°C for 5 h, 23 nL of compounds dissolved in DMSO, positive and negative controls were transferred to the assay plates using a Pintool station. The assay plates were incubated at 37°C for 40 h. The fluorescence and luminescence intensities

were measured at 0, 8, 16, 24, 32, and 40 h time points using a ViewLux plate reader (PerkinElmer) during compound treatment.

HSE, ARE, PPAR-gamma, and p53 β-lactamase (bla) reporter gene assays: HSE-*bla* Hela, ARE-*bla* HepG2, PPAR gamma-UAS-*bla* 293H, and p53-*bla* HCT-116 cells were dispensed at 1500, 2000, 3000, and 4000 respectively in 6 μL/well and 5 μL/well (PPAR-gamma antagonist mode) in 1536-well tissue culture treated black wall/clear bottom plates (Greiner Bio-One) using a Multidrop Combi dispenser. After the assay plates were incubated at 37°C for 5-6 h (ARE-*bla*, HSE-*bla*, PPAR gamma-*bla*, and p53-*bla*) and 18 h (HSE-*bla*), 23 nL of compounds dissolved in DMSO, positive and negative controls were transferred to the assay plates using a Pintool station. For PPAR-gamma antagonist mode, 1 μL/well agonist (50 nM Rosiglitazone) or assay medium was added to each well using an FRD. The assay plates were incubated for 5 h (HSE-*bla*), 16 h (ARE-*bla* and p53-*bla*), and 17 h (PPAR gamma-*bla*) at 37°C. For β-lactamase assay, 1 μL/well LiveBLAzer™ FRET-B/G CCF4-AM substrate (Life Technologies) detection mix was added to each well using an FRD. After 2 h incubation at room temperature, the fluorescence intensity was measured using an Envision plate reader (PerkinElmer) at 405 nm excitation, 460 and 530 nm emissions. For cell viability assay, 3-4 μL/well CellTiter-Glo® Luminescent Cell Viabillity reagent (Promega) was added to each well using an FRD. After 0.5 h incubation at room temperature, the luminescence intensity was measured using a ViewLux plate reader (PerkinElmer). Data were represented as relative fluorescence units (β-lactamase assay; final data expressed as the ratio of 460/530 nm emission values) and relative luminescence units (cell viability assay).

Mitochondrial Membrane Potential (MMP) assay: The MMP assay fluorescence readout measures changes in mitochondrial membrane potential. HepG2 cells were dispensed at 2000

cells/5 μL/well in 1536-well tissue culture treated black wall/clear bottom plates (Greiner Bio-One) using a Multidrop Combi dispenser.[4] After the assay plates were incubated at 37°C for 18 h, 23 nL of compounds dissolved in DMSO, positive and negative controls were transferred to the assay plate using a Pintool station. The assay plates were incubated for 1 h at 37°C. Then 5 μL/well of Mito-MPS dye loading solution (Codex BioSolutions, Inc. Gaithersburg, MD) was added to each well using and FRD. After 0.5 h incubation at 37°C, the fluorescence intensity (490 nm excitation and 535 nm emission for green fluorescent monomers, and 540 nm excitation and 590 nm emission for red fluorescent aggregates) was measured using an Envision plate reader. Data were represented as relative fluorescence units (final data expressed as the ratio of 590/535 nm emission values).

Data Analyses: The new wAUC metric has more compact scale of values due to its normalization relative to the infinite dilution concentration, to enable comparisons at alternate exposure concentration ranges/units. Decreasing effects were flagged for cytotoxicity if the POD value of the effect was not more potent than the POD of cytotoxicity from an assay's counter-screen. Also, the effects identified in the β-lactamase assays were flagged if the responses in the reporter gene channel readout were not matched with the responses after normalization to background. Analysis of compound concentration–response data was performed as previously described Huang 2016.[5] Briefly, raw plate reads for each titration point were first normalized relative to the positive control compound (-100% for antagonist mode and 100% for agonist mode) and DMSO-only wells (0%) as follows: % Activity = $((V_{compound} - V_{DMSO})/(V_{pos} - V_{DMSO})) \times 100$, where $V_{compound}$ denotes the compound well values, V_{pos} denotes the median value of the positive control wells, and V_{DMSO} denotes the median values of the DMSO-only wells, and then corrected by applying a NCATS in-house pattern correction algorithm.[6] Concentration–response titration points for each

compound were fitted to a four-parameter Hill equation yielding concentrations of half-maximal inhibitory activity (IC_{50}) or half-maximal stimulatory activity (EC_{50}) and maximal response (efficacy) values.[7] Compounds were designated as Class 1–4 according to the type of concentration–response curve observed.[5,8]

Noise level (threshold, THR) as the Curvep input: Curvep, a response noise filtering algorithm, was used to process the curves. Curvep relies on user-defined thresholds such as the baseline noise threshold (THR) and the maximum curve deviation (MXDV) to filter the response noise. Among thresholds, it is known that the THR has direct and significant impact on defining activity of testing chemicals.

As the THR is a user-defined parameter, we think that the optimal THR should reflect the intrinsic response variation in the screening data of the substances and has the meaning of minimum response threshold. To achieve this, we iteratively applied Curvep using various THRs on the simulated curves derived from the screening data of the substances, and the optimal THR was identified as the lowest THR at which the variance in potency estimation was sufficiently reduced.[9]

The specific details are explained as follows:

Curve simulation: The response of a curve for each substance was calculated using the Equation 1, where $\widehat{\boldsymbol{\beta_0}}$ and $\widehat{\boldsymbol{\beta_1}}$ were estimated from the linear regression with response as dependent variable and concentration as independent variance from the screening data and $\boldsymbol{\varepsilon}$ is the randomly generated noise

$$y = \widehat{\beta_0} + \widehat{\beta_1}x_1 + \varepsilon$$

The noise was generated from bootstrapping responses from a normal distribution with mean = 0 and SD = standard deviation of responses from vehicle control-only wells. In total, 100 curves

were created for each substance in every readout. For each readout, to identify the optimal THR for either increased or decreased effect, THR from 5% to 95% with an increment of 5% was applied on the simulated curves. Then, the potency/concentration at x threshold was reported (x is 5% to 95% with an increment of 5%) for each simulated curve. For inactive curve (i.e., all responses = 0 after Curvep), the potency was fixed to the maximum tested concentration. The pooled variance of potency of all substances for each THR was calculated. The optimal THR was considered as the lowest THR at which the variance in potency estimation was sufficiently reduced and stabilized. The implementation for identification of the optimal THR is available in the R package, Rcurvep (github.com/moggces/Rcurvep, v0.3.1)

Rcurvep method and metric*:* Curvep is a non-parametric algorithm that minimizes the number of corrections needed to restore curve's monotonicity.[10] Corrected data points are then imputed, on log-concentration scale, from the linear splines between the remaining adjacent points. Based on the corrected curve, several metrics are calculated in the corresponding Rcurvep package (github.com/moggces/Rcurvep, v0.3.1), such as: effective concentrations (EC_{xx}) at various relative levels of response, maximal response (E_{max}), slope of the mid-curve (between EC_{25} and EC_{75}), point-of-departure (POD) as the concentration at which significant level (as defined by user-set threshold) of response is reached, response-weighted concentration (wConc), concentration-weighted response (wResp), area-under-curve (AUC), and wAUC, which is AUC weighted by POD and test range. The updated wAUC (as implemented in Rcurvep) is calculated as AUC * (LO – L) / (POD – L) / (HI– L), where LO and HI are log-transformed lowest and highest test doses, and L is a log-dose for infinite dilution (set by default to -24 in log-molar units, as approximation based on Avogadro constant). Among the curves with the same AUC, this wAUC equation gives

higher weight to curves with lower (more potent) POD and narrower test range (lower HI, and higher LO).

Supplemental Tables

Supplemental Data Table 1: Botanical/Dietary test substance identity and procurement inventory.

Sample ID	Test Substance Name	Botanical Group	Formulation	Lot #	Stock Units	Stock mg/mL Equivalents
NCGC00372657-01	Annatto Extract Acid Proof	Annatto	extract	5512CD	mg/ml	7.686
NCGC00372562-01	Annatto Extract D.S.	Annatto	extract	4208CC	mg/ml	10
NCGC00372567-01	Annatto Extract OWD	Annatto	extract	R441	mg/ml	10
NCGC00372760-01	Annatto Food color, 200	Annatto	extract	98131	mg/ml	10
NCGC00372765-01	Annatto Food Color, Acid Proof	Annatto	extract	97781	mg/ml	10
NCGC00372793-01	Annatto Food Color, No. 4	Annatto	extract	97881	mg/ml	10
NCGC00372615-01	Annatto Oil Soluble	Annatto	extract	4491CC	mg/ml	10
NCGC00372875-01	Annatto Powder (Bixin)	Annatto	extract	98481	mg/ml	10
NCGC00372880-01	Annatto Powder, Type 17.5G	Annatto	extract	98921	mg/ml	10
NCGC00372870-01	Ultrabix Annatto Powder	Annatto	extract	831AZ	mg/ml	10
NCGC00372666-01	Bixin	Annatto	constituent	40F26	mg/ml	10
NCGC00372805-01	Bixin	Annatto	constituent	UJ0945	mg/ml	10
NCGC00372572-01	Black Walnut Extract/Juglone	Black Walnut	extract	010013	mg/ml	10
NCGC00372577-01	Black Walnut Extract/Juglone	Black Walnut	extract	1003-9377	mg/ml	10
NCGC00372647-01	Black Walnut Extract/Juglone	Black Walnut	extract	001784	mg/ml	10
NCGC00372770-01	Black Walnut Extract/Juglone	Black Walnut	extract	145091800	mg/ml	10
NCGC00372784-01	Black Walnut Extract/Juglone	Black Walnut	extract	9300	mg/ml	10
NCGC00372566-01	Cedarwood Oil	Cedarwood Oil	oil	T122303DP	mg/ml	10
NCGC00372656-01	Cedarwood Oil	Cedarwood Oil	oil	T110603JB	mg/ml	10
NCGC00372571-01	Chitosan	Chitosan	constituent	02-CLVB-0760	mg/ml	10
NCGC00372661-01	Chitosan	Chitosan	constituent	02-ASSF-0715	mg/ml	10
NCGC00372570-01	Citral	Citral	constituent	TG6930PG	mg/ml	10
NCGC00372576-01	Citral	Citral	constituent	04402AQ	mM	3.09
NCGC00372646-01	Citral	Citral	constituent	7924-85	mg/ml	10
NCGC00372651-01	Citral	Citral	constituent	M121084	mg/ml	10
NCGC00372775-01	Citral	Citral	constituent	04402AQ	mM	3.09
NCGC00372575-01	Comfrey Root	Comfrey Root	extract	105888	mg/ml	10
NCGC00372764-01	Comfrey Root	Comfrey Root	extract	B020703AP	mg/ml	10
NCGC00372560-01	Corn oil	Corn oil	oil	065K0077	mg/ml	10
NCGC00372769-01	Corn oil	Corn oil	oil	46006-4	mg/ml	10
NCGC00372565-01	Echinacea purpurea, ext.	Echinacea purpurea	extract	0601-6233	mg/ml	10
NCGC00372569-01	Ginkgo Biloba Extract	Ginkgo Biloba	extract	001003	mg/ml	10
NCGC00372578-01	Ginkgo Biloba Extract	Ginkgo Biloba	extract	020703	mg/ml	10
NCGC00372799-01	Ginkgo Biloba Extract	Ginkgo Biloba	extract	GBE-50-001003	mg/ml	2.73
NCGC00372768-01	Ginkgo Biloba Leaves Powder	Ginkgo Biloba	extract	8K13-2C	mg/ml	10
NCGC00372682-01	Kaempferol	Ginkgo Biloba	constituent	142445	mg/ml	10
NCGC00372887-01	Kaempferol	Ginkgo Biloba	constituent	142445	mM	5.7
NCGC00372674-01	Quercetin	Ginkgo Biloba	constituent	049K1532	mg/ml	10
NCGC00372771-01	Quercetin	Ginkgo Biloba	constituent	969-0483-18BL	mg/ml	10
NCGC00372884-01	Quercetin	Ginkgo Biloba	constituent	969-3790-05	mg/ml	10
NCGC00372758-01	Goldenseal	Goldenseal	extract	9806545	mg/ml	10
NCGC00372789-01	Goldenseal	Goldenseal	extract	007-090200	mg/ml	10
NCGC00372881-01	Goldenseal	Goldenseal	extract	HYCA10/7-10.28.01-	mg/ml	10
NCGC00372559-01	Goldenseal Root Powder	Goldenseal	extract	019-0308	mg/ml	10
NCGC00372568-01	Goldenseal Root Powder	Goldenseal	extract	038-0508	mg/ml	10
NCGC00372773-01	Goldenseal Root Powder	Goldenseal	extract	8147	mg/ml	10
NCGC00372788-01	Goldenseal Root Powder	Goldenseal	extract	9025	mg/ml	10
NCGC00372798-01	Goldenseal root powder	Goldenseal	extract	9811518	mg/ml	10
NCGC00372866-01	Goldenseal Root Powder	Goldenseal	extract	9264	mg/ml	10
NCGC00372744-01	Berberine chloride	Goldenseal	constituent	119H0687	mM	7.47
NCGC00372573-01	Grape Seed Extract	Grape Seed	extract	8393-JO	mg/ml	10
NCGC00372763-01	Grape Seed Extract	Grape Seed	extract	UNGS-010201	mg/ml	10
NCGC00372778-01	Grape Seed Extract (ActiVin GSE-2000)	Grape Seed	extract	2001082704F	mg/ml	10
NCGC00372785-01	Grape Seed Extract (ActiVin GSE-2000-S)	Grape Seed	extract	2001032902F	mg/ml	10
NCGC00372672-01	Gum Guggul Extract	Gum Guggul	extract	07172009	mg/ml	10
NCGC00372677-01	Gum Guggul Extract	Gum Guggul	extract	G51177/H	mg/ml	10
NCGC00372777-01	Gum Guggul Extract	Gum Guggul	extract	04172009	mg/ml	10
NCGC00372780-01	Gum Guggul Extract	Gum Guggul	extract	G60493/H	mg/ml	10
NCGC00372874-01	Gum Guggul Extract 10%	Gum Guggul	extract	SBL-S050750	mg/ml	10
NCGC00372558-01	Gugulipid 2.5% (40 Mesh)	Gum Guggul	extract	G100707	mg/ml	10
NCGC00372563-01	Gugulipid 2.5% Granules	Gum Guggul	extract	G51177/H	mg/ml	10
NCGC00372772-01	Gugulipid SCF Extract	Gum Guggul	extract	G80338	mg/ml	10
NCGC00372676-01	Kava Kava Extract	Kava Kava	extract	2638-22599-C	mg/ml	10
NCGC00372687-01	Kava Kava Extract	Kava Kava	extract	082203	mg/ml	10
NCGC00372762-01	Kava Kava Extract	Kava Kava	extract	562	mg/ml	10
NCGC00372779-01	Kava Kava Extract	Kava Kava	extract	9077SDK/COSMO	mg/ml	10
NCGC00372790-01	Kava Kava Extract	Kava Kava	extract	9077SDK/COSMO	mg/ml	10
NCGC00372879-01	Kava Kava Extract	Kava Kava	extract	930056	mg/ml	10
NCGC00372776-01	Milk Thistle Seed	Milk Thistle	extract	12238	mg/ml	10
NCGC00372671-01	Milk Thistle Extract	Milk Thistle	extract	M-069902	mg/ml	10
NCGC00372680-01	Milk Thistle Extract	Milk Thistle	extract	118 is	mg/ml	10
NCGC00372781-01	Milk Thistle Extract	Milk Thistle	extract	MTSXP 895	mg/ml	10
NCGC00372796-01	Milk Thistle Extract	Milk Thistle	extract	B120799KLH	mg/ml	10
NCGC00372876-01	Milk Thistle Extract	Milk Thistle	extract	488081999	mg/ml	10
NCGC00372683-01	Silybin	Milk Thistle	constituent	044K0845	mM	9.75
NCGC00372791-01	Silybin	Milk Thistle	constituent	08212KQ	mg/ml	10
NCGC00372886-01	Silybin	Milk Thistle	constituent	044K0845	mM	10
NCGC00372685-01	Olive oil	Olive oil	oil	L1444RH1000	mg/ml	10
NCGC00372670-01	Pine Bark Extract	Pine Bark	extract	UNPB-10520B	mg/ml	10
NCGC00372757-01	Pine Bark Extract (Pycnogenol)	Pine Bark	extract	B/420	mg/ml	10
NCGC00372669-01	Resveratrol	Resveratrol	constituent	18090-719	mg/ml	10
NCGC00372786-01	Resveratrol	Resveratrol	constituent	231AD	mg/ml	10
NCGC00372869-01	Resveratrol	Resveratrol	constituent	156AB	mg/ml	10
NCGC00372678-01	Safflower oil	Safflower oil	oil	SC-000486	mg/ml	10
NCGC00372761-01	Safflower oil	Safflower oil	oil	MKBB7664	mg/ml	10
NCGC00372797-01	Turmeric	Turmeric	extract	TUP/405B	mg/ml	10
NCGC00372872-01	Turmeric	Turmeric	extract	90H73	mg/ml	10
NCGC00372877-01	Turmeric	Turmeric	extract	46006-4	mg/ml	7.4
NCGC00372792-01	Turmeric oleo resin	Turmeric	extract	2558-A	mg/ml	10
NCGC00372845-01	Curcumin	Turmeric	constituent	46006-4	mM	7.4

Supplemental Figures

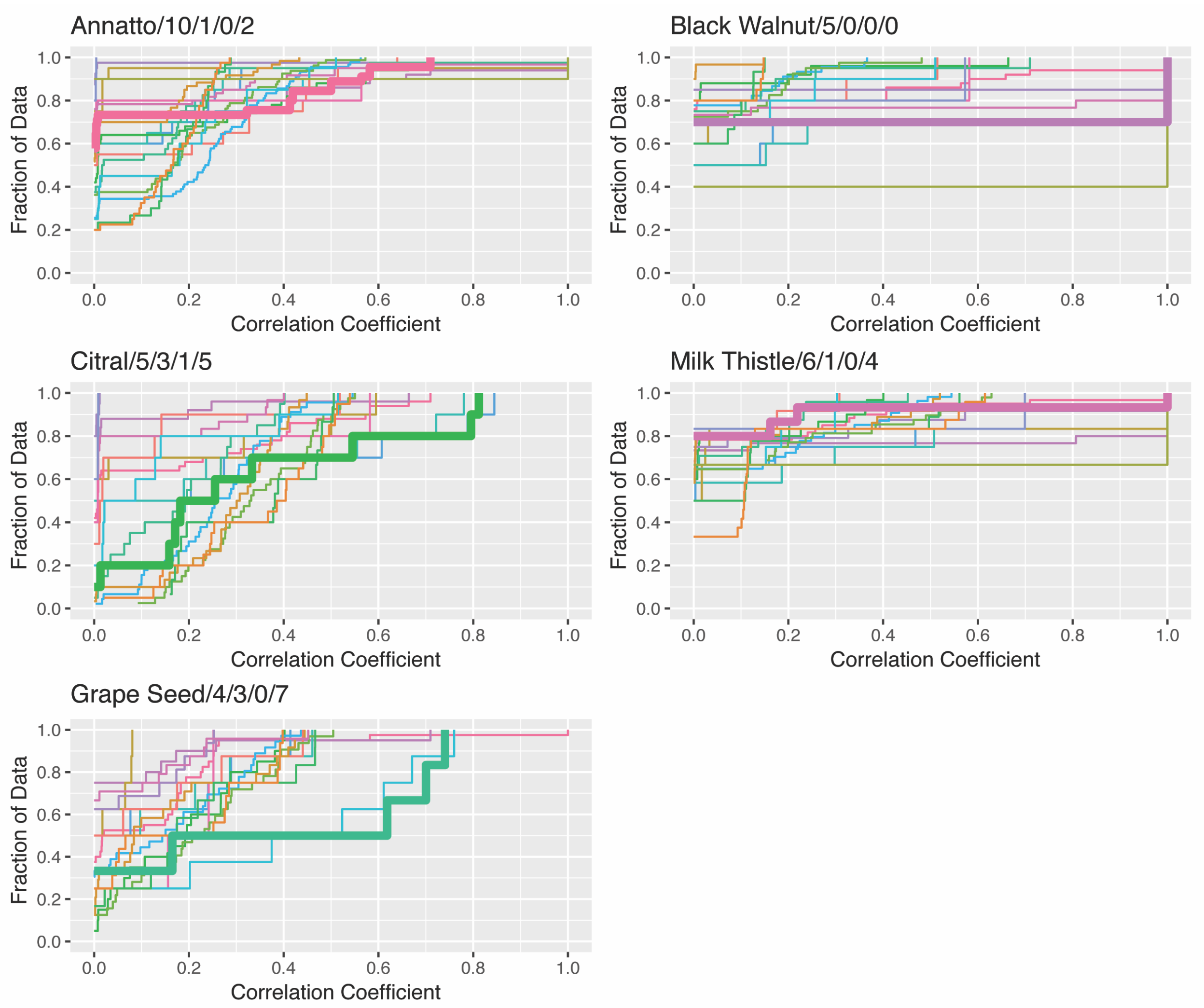

Supplemental Figure 1: Assessment of intra/inter-botanical supplement activity trends. Comparison of botanical/dietary supplements (those with ≥3 different test lots) by Pairwise Spearman rank correlations using responses (wAUC) in all assay readouts. Plots are labeled by botanical group (Annatto, Black Walnut Extract, Citral, Milk Thistle Extract, and grape seed extract) followed by four numbers indicating number of lots assessed/average number of active assays across lots/minimum activity by single lot/maximum activity by a single lot. The distribution of the thick line across the x-axis represents the degree of similarity within a group. The degree of overlap between the thick line and the thin lines represents the uniqueness of the group relative to other tested botanical/dietary supplements.

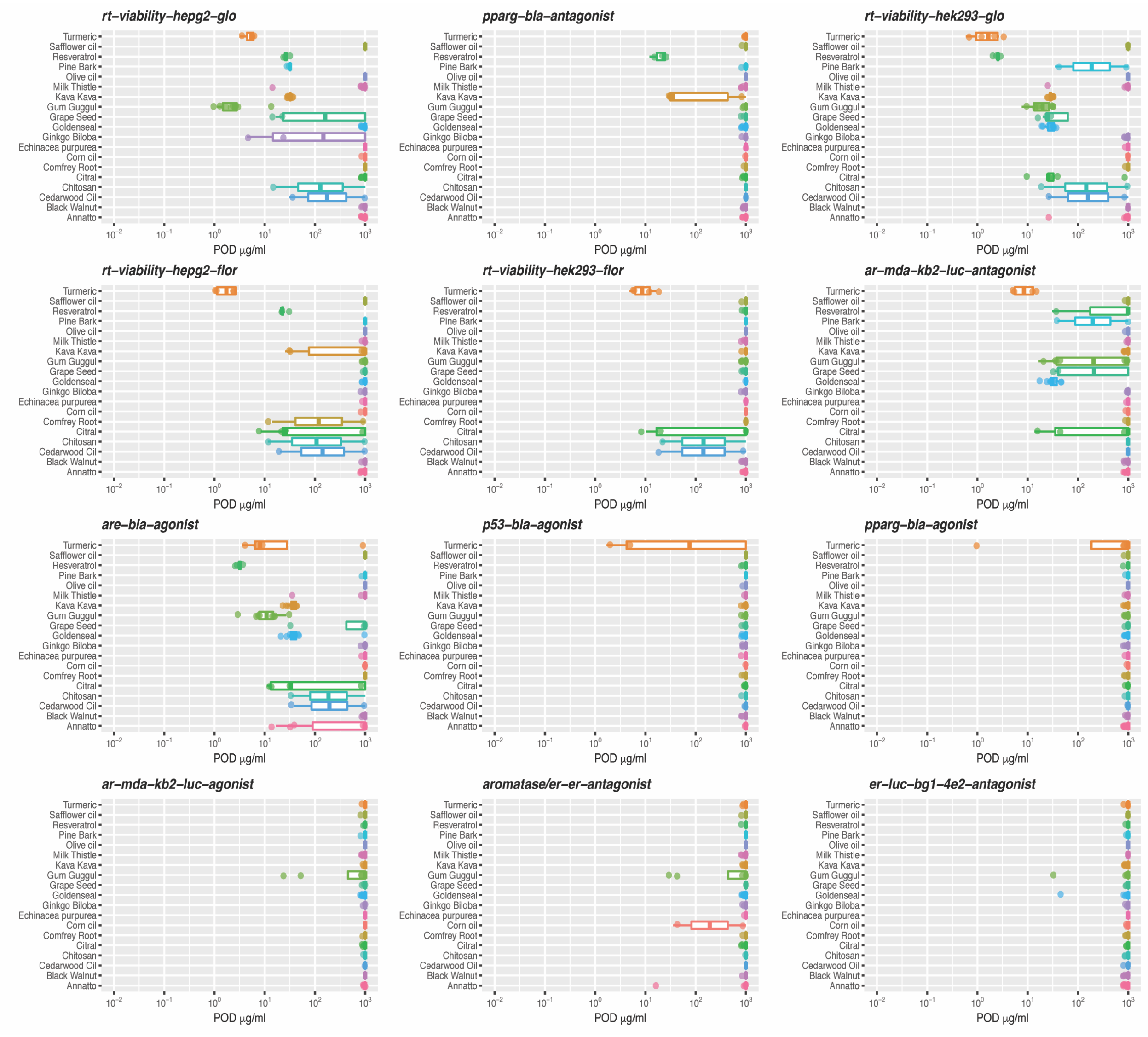

Supplemental Figure 2: Box-plots summarizing relative point of departures (POD) data in the bottom fourteen ranked assays (based on F-value) across tested botanical groups. Note: Two assays are unable to be plotted due to observed inactivity in all tested botanical groups.

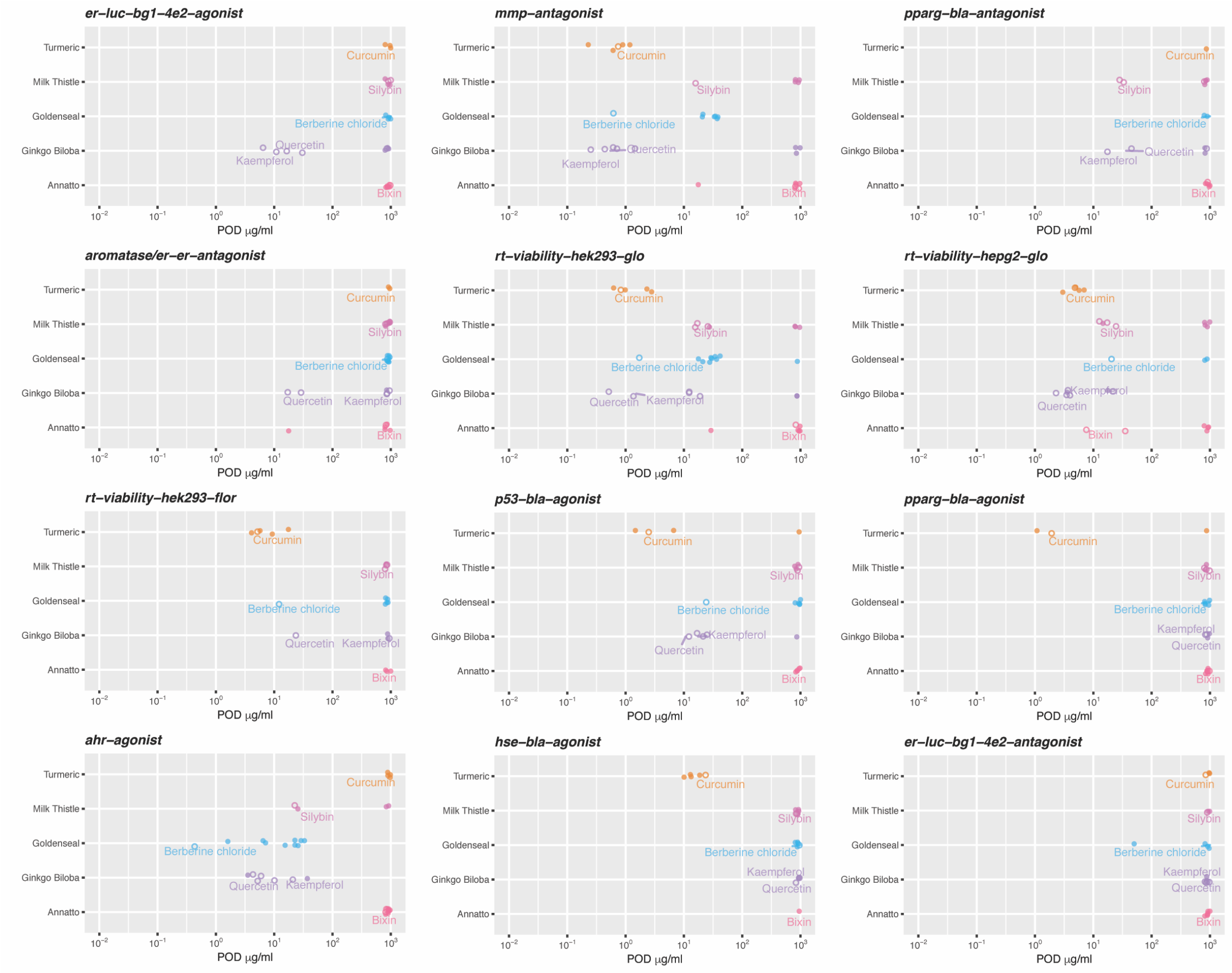

Supplemental Figure 3: Box-plots summarizing relative point of departures (POD) data in the bottom fourteen ranked assays (based on F-ratio) across select botanical groups and associated marker constituents. Note: Two assays are unable to be plotted due to observed inactivity in all tested botanical/constituent groups.

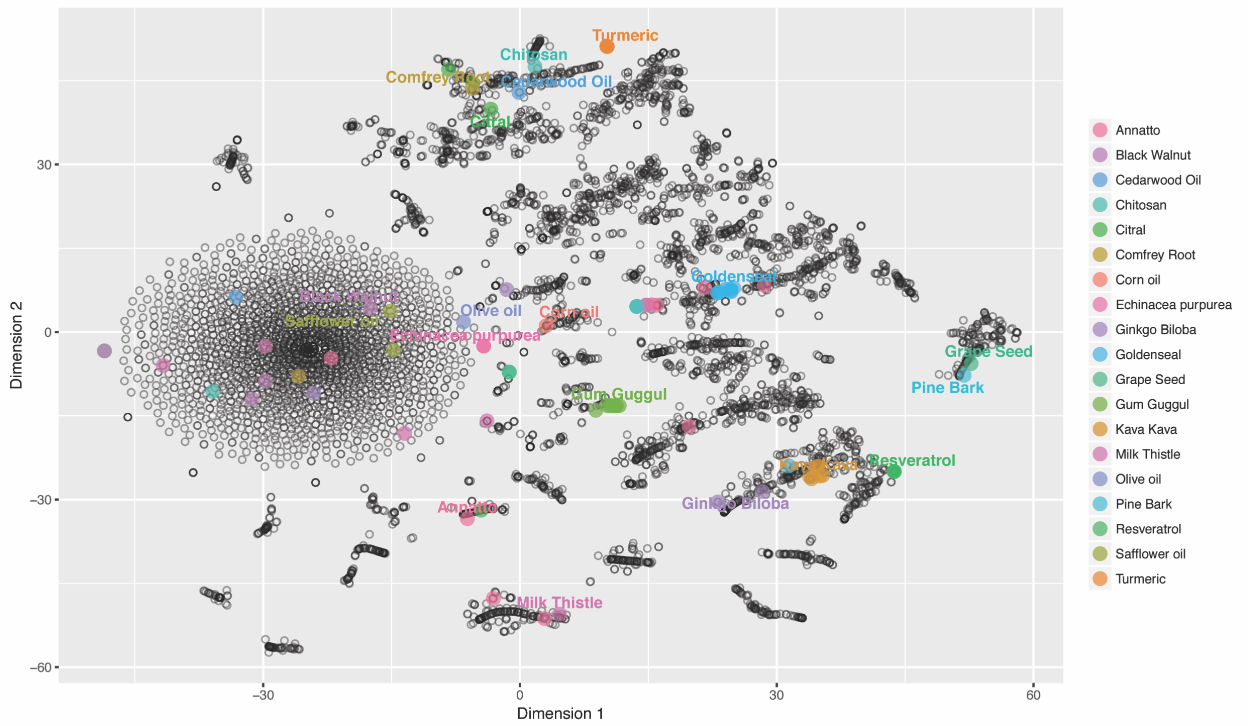

Supplemental Figure 4: tSNE clustering summary of Tox21 library chemical and botanical/dietary extracts based on the responses (wAUC) in all assay readouts. The most active test lot within each botanical group is labeled.

Supplemental Citations

1. Fox, J. T. *et al.* High-throughput genotoxicity assay identifies antioxidants as inducers of DNA damage response and cell death. *Proc. Natl. Acad. Sci.* **109,** 5423–5428 (2012).

2. Chen, S. *et al.* Cell-based high-throughput screening for aromatase inhibitors in the Tox21 10K library. *Toxicol. Sci.* **147,** 446–457 (2015).

3. Hsieh, J. H. *et al.* Real-time cell toxicity profiling of Tox21 10K compounds reveals cytotoxicity dependent toxicity pathway linkage. *PLoS One* **12,** 1–19 (2017).

4. Sakamuru, S. *et al.* Application of a homogenous membrane potential assay to assess mitochondrial function. *Physiol. Genomics* **44,** 495–503 (2012).

5. Huang, R. A Quantitative High-Throughput Screening Data Analysis Pipeline for Activity Profiling. in *High-Throughput Screening Assays in Toxicology* (ed. Xia, H. Z. and M.) 1473 (Humana Press, 2016).

6. Wang, Y. and H. R. Correction of Microplate Data from High-Throughput Screening. In: High-Throughput Screening Assays in Toxicology. in *Methods in Molecular Biology* (ed. H. Zhu and M. Xia) 123–134 (Springer, 2016).

7. Wang, Y., Jadhav, A., Southal, N., Huang, R. & Nguyen, D.-T. A grid algorithm for high throughput fitting of dose-response curve data. *Curr. Chem. Genomics* **4,** 57–66 (2010).

8. Inglese, J. *et al.* Quantitative high-throughput screening: A titration-based approach that efficiently identifies biological activities in large chemical libraries. *Proc. Natl. Acad. Sci.* **103,** 11473–11478 (2006).

9. Hsieh, J.-H. Accounting Artifacts in High-Throughput Toxicity Assays. In: High-Throughput Screening Assays in Toxicology. in *Methods in Molecular Biology* (ed. Xia,

M. and Zhu, H.) 143–152 (Springer, 2016).

10. Sedykh, A. CurveP Method for Rendering High-Throughput Screening Dose-Response Data into Digital Fingerprints. In: High-Throughput Screening Assays in Toxicology. in *Methods in Molecular Biology* (ed. Zhu H. and Xia M.) 135–141 (2016).
